# Diverse drug-resistance mechanisms can emerge from drug-tolerant cancer persister cells

**DOI:** 10.1038/ncomms10690

**Published:** 2016-02-19

**Authors:** Michael Ramirez, Satwik Rajaram, Robert J. Steininger, Daria Osipchuk, Maike A. Roth, Leanna S. Morinishi, Louise Evans, Weiyue Ji, Chien-Hsiang Hsu, Kevin Thurley, Shuguang Wei, Anwu Zhou, Prasad R. Koduru, Bruce A. Posner, Lani F. Wu, Steven J. Altschuler

**Affiliations:** 1 Department of Pharmaceutical Chemistry, University of California, San Francisco, San Francisco, California 94158, USA; 2 Green Center for Systems Biology, University of Texas Southwestern Medical Center, Dallas, Texas 75390, USA; 3 Department of Biochemistry, University of Texas Southwestern Medical Center, Dallas, Texas 75390, USA; 4 Department of Pathology, University of Texas Southwestern Medical Center, Dallas, Texas 75390, USA

## Abstract

Cancer therapy has traditionally focused on eliminating fast-growing populations of cells. Yet, an increasing body of evidence suggests that small subpopulations of cancer cells can evade strong selective drug pressure by entering a ‘persister' state of negligible growth. This drug-tolerant state has been hypothesized to be part of an initial strategy towards eventual acquisition of bona fide drug-resistance mechanisms. However, the diversity of drug-resistance mechanisms that can expand from a persister bottleneck is unknown. Here we compare persister-derived, erlotinib-resistant colonies that arose from a single, EGFR-addicted lung cancer cell. We find, using a combination of large-scale drug screening and whole-exome sequencing, that our erlotinib-resistant colonies acquired diverse resistance mechanisms, including the most commonly observed clinical resistance mechanisms. Thus, the drug-tolerant persister state does not limit—and may even provide a latent reservoir of cells for—the emergence of heterogeneous drug-resistance mechanisms.

## Abstract (web summary)

Cancer cells that survive initial drug treatment can persist in the presence of drugs. Here, the authors generate persister cells that are resistant to the EGFR tyrosine kinase inhibitor erlotinib and show by single cell analysis that multiple mechanism give rise to the drug-resistant persister state.

## Graphical abstract

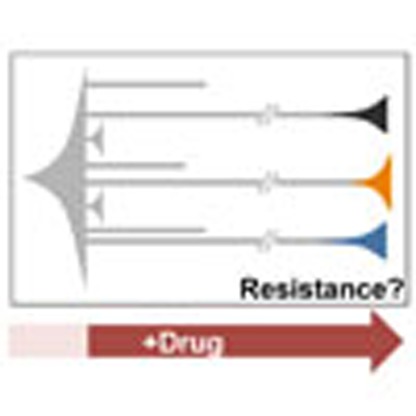

The emergence of diverse resistance mechanisms to targeted therapy is one of the foremost challenges in cancer today^1^. Within the same patient or even tumour, multiple mechanisms of drug resistance can coexist^2^,^3^,^4^. Random, resistance-conferring genetic events preceding drug treatment are an unquestionable means by which this diversity can occur^5^,^6^,^7^. Yet, identifying alternate routes by which cancer cell populations can arrive at resistance mechanisms is of key interest.

One recently proposed alternative route for acquiring resistance is via a drug-tolerant persister state^8^,^9^,^10^,^11^,^12^,^13^. Across multiple cell lines, in response to a variety of strong drug challenges, small subpopulations of cells have been reported to survive by initially entering a drug-tolerant (so-called) ‘persister' state in which there is little-to-no population growth^8^,^12^. Crucially, after long-term treatment (weeks to months) in drug without appreciable growth, a fraction of persisters gain the ability to expand in drug. It has been hypothesized, but never demonstrated experimentally, that survival and expansion through a drug-tolerant state could be part of an initial strategy that mediates the acquisition of bona fide, genetically driven, resistance mechanisms^8^. However, the diversity of resistance mechanisms compatible with evolution from (or through) a persister bottleneck is unclear. Previous work^8^ examined pooled populations of drug-tolerant cells expanded from persisters and did not address this question. Does passage through the persister bottleneck in drug force cells into a single genetic/epigenetic state, or can multiple genetic resistance mechanisms eventually emerge (Fig. 1a)?

Here we chose a strategy that allowed us to follow multiple instances of evolution in drug and focus on resistance mechanisms that emerged from a persister state. We began with a with a population of cancer cells of cancer cells that were recently clonally derived. Next, we ‘crashed' the clonal population with a targeted therapy to reveal a small subpopulation of persisters, some of which eventually gained the ability to proliferate in drug. Finally, to avoid growth competition, we expanded surviving colonies in isolation from one another in drug over the course of a year. This process allowed us to establish a panel of 17 persister-derived, drug-resistant colonies that arose from a single cell. We used a combination of large-scale drug screening and whole-exome sequencing to show that our drug-resistant colonies exhibited diverse resistance mechanisms, including ones observed clinically^14^. Our results suggest that the drug-tolerant persister state does not limit—and may even provide a latent reservoir of cells for—the emergence of heterogeneous drug-resistance mechanisms.

## Results

### Drug resistance emerges from drug-tolerant persisters

We chose as our model system the well-studied, epidermal growth factor receptor (EGFR)-addicted non-small cell lung cancer (NSCLC) cell line PC9 (ref. 15) for several reasons. It has been shown that a small fraction of PC9 cells (∼0.5%) can enter a persister state to evade the strong selective pressure of high concentrations of the EGFR inhibitor erlotinib (2.5 μM, ∼100 × IC50)^8^. In addition, known resistance-conferring genetic mutations can serve as a reference against which to evaluate any emergent genetic diversity.

For our study, we followed the previously established procedure with two crucial changes, which allowed us to focus on individual resistance solutions that emerged from persisters (Methods; Supplementary Fig. 1). First, to reduce pre-existing genetic heterogeneity, we established our persisters from a single, short-passage clonal parental cell line, PC9-1 (∼20 doublings from the single, originating cell). Second, to search for diversity not evident from pooled-population studies, we isolated small, recently expanded colonies that emerged ∼2 months after seeding and expanded them in separate culture wells to eliminate growth competition. (Except when noted otherwise, colonies were cultured and assayed in 2.5 μM erlotinib.)

Similar to previous observations^8^, only a small fraction of cells survived drug treatment (2.5 μM erlotinib; Supplementary Fig. 2a); drug-tolerant cells were largely in a state of negligible growth during the first 6 weeks of observation; and drug tolerance could be abolished with co-treatment of erlotinib with trichostatin A (Supplementary Fig. 2b). Of the ∼50 colonies originally isolated, 17 survived the expansion process, which took ∼6–8 months to generate three confluent 10-cm plates each. We refer to these persister-derived erlotinib-resistant colonies as PERCs.

**Figure 1:**
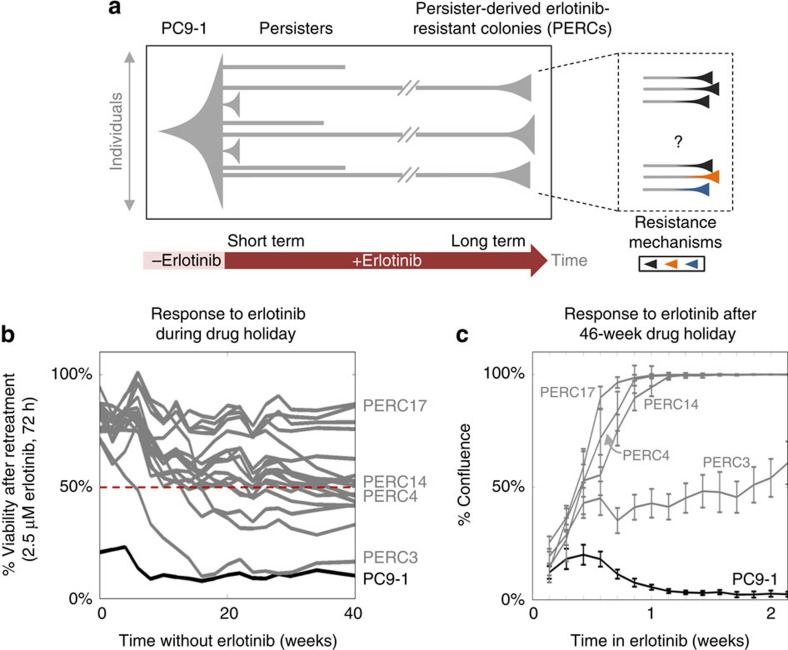
The emergence of persister-derived erlotinib-resistant colonies from PC9-1 cells. (**a**) Schematic outline of the emergence of drug-resistant cancer cell populations (right), originating from a common clone (left), through the bottleneck of drug-tolerant, slow-growing persisters (middle grey lines). The vertical axis indicates population size; the horizontal axis is time. (**b**) Evolution of PERC sensitivity to erlotinib after removal from drug treatment. PERCs were grown in erlotinib-free media and periodically retested over ∼40 weeks for erlotinib sensitivity (2.5 μM erlotinib, 72 h CellTiter-Glo assay; Methods). Black: PC9-1; grey: PERCs. Viability values are calculated as the mean of technical replicates (*n=3*); average s.d. between replicates is 1.23. Dotted red line marks 50% viability as compared with drug-free growth; we note that when a PERC response curve crosses this line it has an IC50 of 2.5 μM erlotinib (which is 100 times the IC50 of PC9). (**c**) Short-term regrowth of PERCs in erlotinib after drug holiday. After ∼46 weeks of growth in erlotinib-free media, selected PERCs were grown in 2.5 μM erlotinib and imaged daily for 2 weeks. Growth is quantified in terms of percentage of field of view covered by cells (Methods). For each cell line, at each time point, 484 images were analysed and the mean fraction of cellular area calculated is reported here (error bars denote s.d. across the 484 images). A typical single cell covers ∼0.05% of the field of view as defined.

We tested whether our isolated PERCs were in the previously described, reversible state of drug resistance^8^. A functional signature of this state is eventual reversion to erlotinib sensitivity after an extended (30 passages) ‘drug holiday'^8^ (as opposed to non-reverting resistance due to clonal genetic events^16^). We continuously cultured the 17 PERCs in erlotinib-free media for over 40 weeks and periodically retested for erlotinib sensitivity (Fig. 1b; for our PERCs, one passage ≈1 week; Methods). During the long-term drug holiday, we observed that nearly all of the PERCs remained considerably more resistant to erlotinib than PC9, although to varying degrees. (We note that 50% viability of PERCs at 2.5 μM (Fig. 1b, red line) implies, by definition, an IC50 of 2.5 μM erlotinib, which is 100 × the IC50 of PC9; thus, 50% viability in this assay implies strong drug resistance compared with PC9.) To better understand the one apparent exception, PERC3, we used an image-based assay to profile PERC growth kinetics in erlotinib over a longer time period (2 weeks in Fig. 1c versus 72 h in Fig. 1b). As expected, PC9-1 cells experienced widespread cell death after treatment with erlotinib before settling into a persister state (∼9–14 days) characterized by drug tolerance and negligible growth (Fig. 1c and Supplementary Fig. 2a,c). In contrast, all tested PERCs grew in drug; the apparent reversion to sensitivity of PERC3 simply reflected a relatively slower, but net positive growth rate. As our PERCs appeared not to have reverted to the original level of PC9-1 erlotinib sensitivity, we wondered what drug-resistance mechanisms they had acquired.

### Evidence for diverse vulnerabilities via drug screening

To investigate erlotinib-resistance mechanisms present in our 17 PERCs, we performed a large-scale drug screen^17^. This allowed us to scan for therapeutic vulnerabilities among our PERCs that were absent in PC9-1, and thereby identify pathway or target alterations that conferred resistance. We assayed the sensitivities of our PERCs to a panel of 560 anticancer compounds in combination with erlotinib (Methods; Supplementary Data 1; note that PC9-1 cells were assayed against the drug library in erlotinib-free media, as treatment with erlotinib potently kills these cells). To search broadly for potential vulnerabilities, the panel contained a diverse collection of compounds, including drugs targeted to the specific erlotinib-resistance-conferring T790M-EGFR mutation^18^,^19^,^20^,^21^, kinase inhibitors affecting multiple cancer-related pathways and chemotherapy and epigenetic drugs. Each compound was assayed over a sixfold dosage range, in duplicate and for all 17 PERCs and control PC9-1.

**Figure 2:**
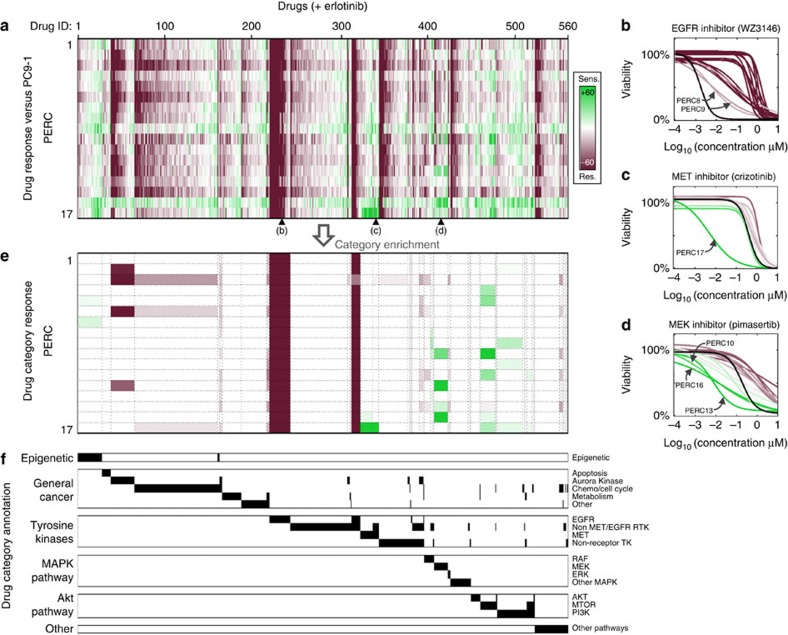
Identification of PERC drug-resistance mechanisms via drug screening for therapeutic vulnerabilities. Response of PERCs versus PC9-1 to a diverse drug library. (**a**) Heatmap: drug-response scores of the PERCs (screen performed in erlotinib-containing media) relative to PC9-1 (screen performed in drug-free media). Rows: PERCs. Columns: 560 anticancer compounds. Scores (green/red colours): based on signed-area differences between drug-response curves of PERCs versus PC9-1 (Methods). Each response score reflects six doses performed in duplicate (*n=*2). (**b**–**d**) Shown are smoothed response curves with respect to selected drugs (corresponding to black triangles in **a**). Graphs: PERCs (drug+2.5 μM erlotinib; response curves coloured according to scores) versus PC9-1 (only drug no erlotinib; black). Green/red: drug-response scores of PERC compared with PC9-1. Smoothed curves were constructed by fitting the mean viability (*n*=2) at each dose to a sigmoidal function using an unweighted least-squares fit (Methods). (**e**) Heatmap: enrichment of PERCs (rows) for strong response to specific drug categories (columns). Drug-category-response scores are based on a hypergeometric test for varying drug-response scores (Methods; Supplementary Fig. 3). Green/red: colours as in **a**. (**f**) Annotation of drugs (columns) to specific drug categories (rows).

We focused initially on identifying PERCs whose drug responses in combination treatment with erlotinib were strongly altered from PC9-1's drug response without erlotinib. There are a number of approaches to assess drug sensitivity from dose–response curves^17^,^22^,^23^,^24^. Here we chose to compute a sensitivity score based on signed-area differences between drug-response curves of PERCs versus PC9-1 that took into account replicate variability (Fig. 2a–d and Methods; an alternative scoring based only on signed-area differences gave similar results, Supplementary Figs 5–7). The large-scale drug screen allowed us to interrogate drug-resistance mechanisms across our collection of PERCs. A few broad trends were noticeable. As compared with PC9-1, PERCs were generally resistant to EGFR inhibitors (as might be expected, for example, Fig. 2b), Aurora kinase inhibitors and chemotherapeutics. Further, some PERCs developed broad resistance (for example, PERC3) or sensitivity (for example, PERC16) to drugs belonging to multiple drug classes (Fig. 2e,f).

We next searched for functional evidence of PERC resistance/vulnerability in different mechanistic drug categories. It is not to be expected that a PERC would respond similarly to every drug within the same category. Therefore, we developed a drug-category-response score to search within each defined category for evidence of PERC sensitivities to a larger-than-expected fraction of drugs (Fig. 2e and Supplementary Fig. 3; scores were normalized per PERC across all drugs; Methods). There was no single category for which all 17 PERCs were vulnerable. However, we identified specific vulnerabilities of (Fig. 2c–f): PERC17 to MET drugs (including SGX-523, INCB28060, JNJ-38877605 and crizotinib); PERCs 10, 13 and 16 to MEK inhibitors (including selumetinib, PD0325901 and pimasertib); and PERCs 4, 5, 10, 12 and 17 to mechanistic target of rapamycin (MTOR) drugs (including rapamycin and everolimus). Taken together, our drug screen identified putative, mechanistically distinct vulnerabilities, suggesting that our PERCs evolved multiple strategies to escape erlotinib treatment.

### Evidence for diverse resistance mechanisms via sequencing

**Figure 3:**
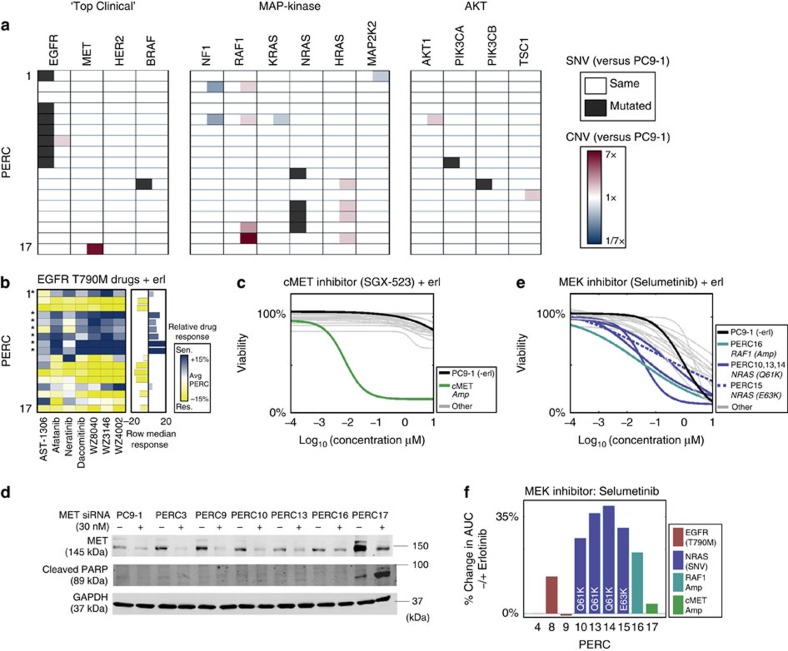
Assessment of PERC drug-resistance mechanisms via genetics and specific perturbations. (**a**) Comparison of genetic alterations in PERCs (rows) for selected genes implicated in erlotinib resistance (columns). Black: presence of a non-synonymous single-nucleotide variant (SNV) versus PC9-1; red/blue: CNVs corresponding to amplification/deletions of genetic regions (Methods) versus PC9-1. (**b**–**f**) Corroboration of genetic information with predicted vulnerabilities. (PERCs were in erlotinib media; PC9-1 cells were in erlotinib-free media). (**b**) Response among PERCs to drugs targeting the EGFR T790M mutation: (left) heatmap: relative response of PERCs (rows) to EGFR T790M drugs (columns). Drug response (blue/yellow) is assessed by change in the AUC of a PERC from the mean AUC among all PERCs (Methods). (Right) Bar plot: row median response across drugs. *: PERCs with EGFR T790M mutation. (**c**) PERC dose–response curves to SGX-523, targeting cMET. Colours: PC9-1 (black); cMET-amplified PERC17 (green); other PERCs (grey). Curves are generated as in Fig. 2b. (**d**) Transient siRNA-mediated knockdown of MET in PC9-1 and a subset of PERCs. MET knockdown in PERC17 (only cell line with MET amplification) induces an increased cleavage of PARP, a marker for apoptosis. GAPDH: loading control. (**e**) PERC dose–response curves to Selumetinib, which targets MEK. Colours: PC9-1 (black); PERCs with mutations relevant to MEK sensitivity (legend); other PERCs (grey). Curves are generated as in Fig. 2b. (**f**) Test for role of MEK as an erlotinib-bypass mechanism. Bars indicate the strength of MEK bypass; height measures the differences in AUC between response curves for Selumetinib (*n*=4) and Selumetinib+erlotinib (*n*=3; six doses as in **e**). Colours: PERCs with mutations in NRAS (blue) and RAF1 (teal), both upstream of MEK; other PERCs (legend).

We next sought to use genetics as a way to corroborate predicted vulnerabilities as well as to identify mechanisms that were not detected by our initial analysis of the drug screen. From our exome-sequencing data, we identified genetic changes between each PERC and the parent PC9-1 (ref. 25; Fig. 3a, Methods; only amplifications >2.5 × compared with PC9-1 are reported below). The derivation of the PERCs from a single, clonal parent offered a unified basis to identify and interpret genetic changes.

We first searched for mechanisms of erlotinib resistance that are most commonly observed in the clinic. On the basis of the sequence data, we found the T790M mutation in EGFR^20^,^21^ present in PERCs 1, 4–9 (Fig. 3a, Supplementary Fig. 5 and Supplementary Table 1). This caused us to re-evaluate our analysis of the drug screen. While all PERCs became more resistant to EGFR drugs when compared with PC9-1, comparison of PERCs with each other revealed that those harbouring a T790M mutation showed increased, albeit partial, sensitivity to T790M-targeting drugs (including afatinib, dacomitinib and WZ3146 (ref. 18); Figs 2b and 3b and Supplementary Figs 4c and 5). In addition, we found genetic evidence for MET amplification^4^,^26^ in PERC17 (Fig. 3a). This observation is consistent with recent findings from Engelman and colleagues^27^ (published whilst our work was under consideration) demonstrating that acquisition of EGFRT790M mutation by drug-tolerant cells may result in a diminished apoptotic response of EGFRT790M targeted drugs. This was confirmed at the single-cell level using fluorescence *in situ* hybridization (Supplementary Fig. 6c). PERC17 exhibited an exquisite sensitivity to MET inhibitors (Figs 2 and 3c and Supplementary Figs 4 and 6a) and apoptosis from short interfering RNA (siRNA) knockdown of MET (Fig. 3d). Thus, MET amplification is a bona fide resistance mechanism for PERC17. To our knowledge, a MET amplification has never previously been reported for the parent PC9 line. Together, T790M and MET have been implicated in over half of all clinically reported cases of EGFR-addicted NSCLC with resistance to first-generation EGFR inhibitors^14^ (for example, erlotinib and gefitinib).

We next examined genetic changes in the MAPK pathway, one of the most frequently mutated pathways associated with erlotinib resistance^28^. We observed point mutations in NRAS for PERCs 10, 13, 14 (Q61K) and PERC15 (E63K), two mutational events that have been implicated in erlotinib resistance in preclinical models^29^ (Fig. 3a and Supplementary Fig. 7). PERC16 exhibited an amplification of RAF1, a genetic alteration that has not been reported in lung cancer but has been characterized as a driver mutation in other cancer types^30^ (Fig. 3a and Supplementary Fig. 7). We used our genetic data to revisit our drug screen, and found that PERCs 10, 13, 16 were sensitive to drugs targeting MEK (for example, selumetinib), which is a downstream member of the MAPK pathway (Figs 2 and 3e and Supplementary Figs 4, 7 and 9)^31^. PERCs 14 and 15 did not display this sensitivity across all drugs in our initial analysis of the drug screen (Fig. 2); however, re-examination of response curves, overlaid with genetic data, revealed that all NRAS and RAF1 mutants had relatively higher MEK sensitivities than the other PERCs (Fig. 3e and Supplementary Fig. 7). Further testing revealed that all PERCs carrying NRAS and RAF1 mutations had higher sensitivity to co-treatment with erlotinib and MEK inhibitors than to either alone, suggesting a role for MEK in ‘bypass' signalling^17^,^32^ (Fig. 3f).

As might be expected, not every putative genetic mechanism was found to correspond to a drug vulnerability. Our drug screen helped to identify which, among multiple genomic alterations harboured by a PERC, might serve as primary drivers of acquired resistance. For example, in PERC9, in addition to the T790M mutation in EGFR, we observed a mutation in PIK3CA (E542K); this mutation is implicated in driving constitutive signalling through AKT^33^, but was not corroborated with drug sensitivities (Figs 2 and 3a). This is consistent with growing evidence that PIK3CA mutations may be bystander mutations in NSCLC^34^. In addition, the mutation in BRAF (G466A) for PERC11 was not identified as a vulnerability with our drug data. Conversely, not every drug vulnerability was found to correspond to an obvious genetic mechanism. For example, we observed mTOR sensitivity in PERC 10 for which we could not find any obvious genetic basis (Figs 2 and 3a). Further, not all NSCLC erlotinib-resistance mechanisms reported in the literature were exhibited by the PERCs; we found no compelling evidence of transformation to small cell lung cancer, epithelial-to-mesenchymal transition or activation of IGF1R, AXL or NFK-B. We were unable to determine the erlotinib-resistance mechanisms for PERCs 2, 3, 11 and 12, suggesting that the diversity of resistance mechanisms compatible with the persister state could be even greater than what we have found. Of these, PERC3 stood out as having nearly threefold more mutations than any other PERC, potentially because of mutation in the DNA polymerase gene PolN (ref. 35; Supplementary Fig. 8). Finally, some of our PERCs (for example, with multiple concurrent genetic alterations and/or drug vulnerabilities) may themselves be heterogeneous, comprising multiple subpopulations with different resistance mechanisms; if true, then there is even more diversity emerging from the persister state than we described. Nevertheless, in total, we discovered pharmacological and/or genetic evidence (as well as corroborating reverse phase protein array (RPPA) data^36^; Methods; Supplementary Figs 5–7) for mechanisms of erlotinib resistance in 13 of our 17 PERCs (Supplementary Fig. 9).

## Discussion

Cancer therapy has traditionally been focused on eliminating fast-growing cells. Here we focused on drug-resistant cancer populations that emerge from a persister state in which cells show little-to-no growth for weeks to months in drug treatment. Our work is a proof-of-principle study that demonstrates for the first time that diverse drug-resistance mechanisms can emerge from persisters, derived from a single, recent ancestor cell and grown under the same selective pressure (Fig. 1a). This heterogeneity presents considerable clinical challenges for ‘personalized' therapy: even if an effective therapy is selected for one PERC, there is no guarantee (and indeed it is not true in our data) that this drug would be effective for other PERCs, which in practice may have been undetected. Persisters, which are a small subpopulation of the bulk cancer population, are currently difficult to study in a clinical setting, and there is no known molecular signature of having passed through this state clinically.

The diversity of resistance mechanisms we observed suggests that passage through the persister state is not a limiting factor in the emergence of drug-resistance heterogeneity (Fig. 1a). Although our study was not focused on when or how resistance arose, we believe it unlikely that our diverse resistance mechanisms were all pre-existing at the time of drug treatment: resistant cells would have had to emerge *de novo* within 20 generations from a single cell without selective pressure and then not expand appreciably for ∼6 weeks in drug. We suspect, as previously conjectured^8^, that persisters provide a drug-tolerant reservoir of cells from which drug-resistance mechanisms can eventually emerge^37^. Our study raises a number of interesting questions. Would different initial cells, passing through the persister bottleneck, give rise to the same resistance landscape? Are persisters themselves in diverse molecular states and, if so, would different persister states favour different sets of resistance mechanisms? These unanswered questions provide motivation for further studies of the timing, diversity and mechanisms by which drug resistance can arise from (or through) the persister bottleneck in different selective pressures and cancer types.

Our work suggests yet a new layer of complexity for treating cancer. Diverse drug-resistance mechanisms can arise from pre-existing mutations before treatment (as has been extensively studied^5^,^6^,^7^) as well as from slow-growing persisters after long-term treatment (which we study here). In fact, it is possible that both mechanisms contribute to drug-resistance heterogeneity in the clinic^8^. Certainly, eliminating, modulating or even anticipating the range of drug-resistance solutions that can emerge from the persister state will help guide the treatment of cancer.

## Methods

### Medium conditions

We made use of two types of media in our experiments. First, ‘erlotinib-free media' is composed of RPMI 1640 (Corning #10 040 CM) supplemented with 5% fetal bovine serum (Life Technologies #16140-071) and 1% Antibiotic-Antimycotic (Life Technologies #15240-062). Second, ‘erlotinib media' is composed of erlotinib-free media and 2.5 μM erlotinib HCl (Selleckchem, Cat.#S1023). Unless otherwise stated, all experiments with PC9 and PC9-1 were performed in ‘erlotinib-free media' and experiments with PERCs were performed in ‘erlotinib media'.

### Generation of clonal cell line PC9-1 in erlotinib-free media

We made use of the ‘EGFR-addicted' NSCLC cell line PC9 acquired from the Minna Laboratory at UT Southwestern; single-nucleotide polymorphism cell line fingerprinting was performed to confirm the cell identity. Overall, 10,000 PC9 cells were seeded on a 10-cm plate. At this low cell density, most cells were isolated from one another. PC9 clonal colonies were selected (we chose colonies that were well separated from others to maximize the chance of being clonally derived) and transferred to a new 6-well plate. These clones were then rapidly expanded from a 6-well plate to one 10-cm plate. The process of generating a confluent plate for each of the clonal populations took ∼2 weeks. Four vials of each clone were frozen down using all cells from the single confluent 10-cm plate. We designated one of these clones PC9-1 and used it for all subsequent experiments.

### Generation of PC9-1-derived PERCs in erlotinib media

PERCs were derived by performing the following steps (Supplementary Fig. 1a). We note that erlotinib media was used for the whole duration of the PERC generation time (∼2 months before isolation and ∼7±1.5 months after isolation) and changed regularly (∼every 2–3 days). Five 10-cm plates were each seeded with 100,000 PC9-1 cells, allowed to adhere overnight and then treated with erlotinib media. Most cells died, leaving a few, isolated, drug-tolerant and slow-growing cells (‘persisters') on the plates. Clearly separated colonies (∼50) were isolated and transferred to 96-well plates between 6 and 8 weeks of drug treatment. Colonies were expanded from 96-well plates to 24-well plates to 6-well plates to one confluent 10-cm plate, and then finally to three confluent 10-cm plates. Plate transfers were performed only when the cells were grown to confluence. Nine vials of these persister-derived erlotinib-resistant colonies (‘PERCs') were frozen down using all cells from the three confluent 10-cm plates. To obtain sufficient cells for our large-scale experiments (drug screening, exome-sequencing and RPPA assays), we expanded the PERCs and PC9-1 cells ∼6 further passages. Cell lines were periodically examined and found negative for mycoplasma contamination during the course of this work.

### Persister time course

PC9-1 cells were plated in triplicate in six-well plates with erlotinib-free media at 40,000 cells per well (Supplementary Fig. 2a). Media was changed to erlotinib media after allowing cells to adhere overnight. The cells were grown for 14 days, with erlotinib media being changed every 3 days. Cells were imaged every 2 days at 10 × magnification with phase-contrast on a Nikon Ti Eclipse.

### Persister drug response

PC9-1 cells were plated in duplicate in six-well plates with erlotinib-free media at 40,000 cells per well (Supplementary Fig. 2b). Media was changed to drug-containing media (described below) after allowing cells to adhere overnight. Plates were treated with various drugs either singly in erlotinib-free media or in combination with erlotinib media. The following drugs were used: 0.1 μM WZ8040 (Selleckchem, Cat.#S1179), 0.1 μM WZ3146 (Selleckchem, Cat.#S1170), 0.1 μM SGX-523 (Selleckchem, Cat.#S1112), 0.0316 μM Crizotinib (PF-02341066; Selleckchem, Cat.#S1068) and 0.02 μM trichostatin A (Selleckchem, Cat.#S1045). One pair of wells was used as controls with no drug in either erlotinib-free media or erlotinib media. The cells were grown for 14 days, with media being changed every 3 days. Cells were imaged every 3 days at × 10 magnification with phase-contrast on a Nikon Ti Eclipse.

### Reversion

Long-term reversion experiments were performed by maintaining our established PERCs in erlotinib-free media. PERCs and PC9-1 were then probed for their responses to erlotinib periodically over the course of 40 weeks (Fig. 1b,c and Supplementary Figs 1b and 2c). We tested for per cent viability (relative to vehicle-treated cells) of PERCs and PC9-1 treated with 2.5 μM erlotinib for 72 h using CellTiter-Glo assays. Bright-field images of PERCs at × 10 magnification were captured daily after long-term (46 weeks) growth in drug-free media and subsequent re-treatment with 2.5 μM erlotinib (Supplementary Fig. 2c. Image analysis was used to identify the portions of these images occupied by cells. A ‘% confluence' for each image was quantified by the fraction of pixels occupied by the identified cellular areas.

### Drug screen

The primary screen (Fig. 2) was performed at the UT Southwestern High-Throughput Screening (UTSW-HTS) Core Facility. For the primary screen, a custom library was constructed using the following libraries: Kinase Inhibitor Screening Library (96-well; Selleckchem, Cat.#L1200), Epigenetic Compound Library (96-well; Selleckchem, Cat.#L1900), Apoptosis Compound Library (96-well; Selleckchem, Cat.#3300), InhibitorSelect 384-Well Protein Kinase Inhibitor Library I (EMD Calbiochem, Cat.#539743, Batch#D00105831) and the NCI Oncology Set (Plates 4,762 and 4,763). Cell lines were each seeded in 384-well plates at an empirically determined optimal seeding density (defined as the seeding density that resulted in vehicle-treated cells being 70–80% confluent at the end of the experiment) and allowed to adhere overnight. Compounds and negative controls were added using a BIOMEK liquid-handling robot on the second day, resulting in a final DMSO concentration of 0.5%, and six, tenfold dilutions of compound doses from 10 μM to 100 pM. Cells were then incubated for 96 h at 37 °C and 5% CO_2_. Next, media was removed and 25 μl of CellTiter-Glo diluted 1:5 with passive lysis buffer (Promega) was added using a Multidrop Reagent Dispenser. Plates were incubated for 10 min at room temperature with shaking and read on an Envision plate reader (Perkin Elmer). Per cent viability calculation was performed by UTSW-HTS using the following formula for single-point normalization: 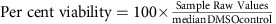.

A small-scale ‘bypass' experiment (Fig. 3f and Supplementary Figs 6b and 7b) was performed at the Small Molecule Discovery Center at UCSF using the same protocol described above. Selected PERCs were treated with ±erlotinib conditions and either SGX-523 (MET drug) or Selumetinib (MEK drug). Drug-response curves were measured at six doses, with multiple technical replicates (*n*=3 +erlotinib; *n=*4 −erlotinib) per drug dose.

### Exome-seq

Genomic DNA was extracted from confluent 15-cm plates using the QIAamp DNA Micro Kit (Qiagen #56304; Fig. 3). The user-developed protocol for ‘Purification of genomic DNA from culture cells using the QIAamp DNA Micro Kit' was followed, except that lysis and ethanol precipitation steps were scaled up twofold, and samples were RNase-treated. Samples were submitted to Beijing Genome Institute (BGI) for quality control, library preparation and whole-exome sequencing. Only samples that were found to be ‘Qualified (A level)' in the quality-control phase were allowed to proceed. ‘Qualified (A level)' samples were defined as samples where (1) the total quantity was over 6 μg; (2) a single band of DNA that was greater than 20 kb with no degradation was detectable using agarose gel electrophoresis; (3) sample concentration was >37.5 ng μl^−1^; and (4) OD_260/280_=1.8–2.0. A 150–200-bp insert library (Agilent SureSelect Human All Exon v4 kit) was used for library construction. Sequencing was performed at BGI on an Illumina HiSeq 2000 sequencer with a paired-end 100-bp read length, and 100 × coverage per sample.

### MET knockdown

The following antibodies were used: MET (Cell Signaling #4560; 1:1,000 dilution), GAPDH (Santa Cruz #sc-47724; 1:3,000 dilution) and Cleaved PARP (Cell Signaling #9541; 1:1,000 dilution; Fig. 3d and Supplementary Fig. 6d–g). Transient knockdown of MET was achieved using SignalSilence MET siRNA I (Cell Signaling #6618), with SignalSilence Control siRNA (Cell Signaling #6568) as a negative control. Cells were seeded in a six-well plate, allowed to adhere overnight and then transfected using Lipofectamine RNAiMax transfection reagent (30 nM siRNA). Whole-cell lysates were collected 72 h post transfection using RIPA buffer supplemented with phenylmethyl sulphonyl fluoride, sodium orthovanadate and a protease inhibitor cocktail (Santa Cruz #sc-24948). SDS–PAGE immunoblots were performed, and data were collected using the LI-COR Odyssey infrared imaging system. Uncropped blots are shown in Supplementary Fig. 6.

### Fluorescence *in situ* hybridization

Cells were harvested and fixed in methanol:acetic acid (3:1) and air-dried slides were prepared (Supplementary Fig. 6c). Probe and nuclear DNA were co-denatured at 72 °C in formamide and hybridized overnight at 37 °C. After washing and counterstaining with 4,6-diamidino-2-phenylindole, analysis was performed using standard fluorescence microscope and images were captured using the Applied Spectral Imaging system. Probes used are CEP-7 (green) and cMET (red; Abbott Molecular, Downers Grove, IL).

### RPPA

Cells were seeded onto six-well plates at a density of 150,000 cells per well and cultured for 24 h (Supplementary Figs 5, 6a and 7a). Cells were lysed using the protocol outlined by the MD Anderson Functional Proteomics Core Facility, where RPPA was performed. Total protein concentration in lysates was determined by performing a BCA assay, and samples were adjusted to a concentration of 1–1.5 μg μl^−1^. Samples were then denatured using the SDS sample buffer recommended by the core facility, boiled for 5 min and then stored at −80 °C before being shipped to MD Anderson on dry ice. Samples were then serially diluted and arrayed onto nitrocellulose-coated slides. Slides were then probed with the core facility's collection of antibodies (listed on website as ‘CoreStdAbList 1_21_2014.xls'), and signal was generated using a 3, 3′-diaminobenzidine colorimetric reaction-based system. Background subtraction and spot density determination were performed using the MiroVigene software. The relative concentration of each protein of interest was defined using the ‘Super Curve Fitting' method developed by MD Anderson's Functional Proteomics Core Facility.

### Drug-response score

We use the notation 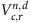 to denote the percentage viability of PERC *n* (=1–17) treated with drug *d* (=1–560) at concentration *c* (=1–6) and replicate measurement *r* (=1–2). We sought to identify PERCs whose drug response differed strongly and reproducibly from that of PC9-1. We made use of the drug response in terms of the area under the curve (AUC) to quantify changes:


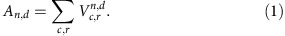


Our score for change reflected the degree to which AUCs for response curves of PC9-1 and PERCs were distinguishable, given a notion of experimental variability. Our measure of variability was constructed by considering the distribution of AUCs when systematically sampling from the replicate measurements; at each of the six concentrations *c*, we chose one of two replicates, giving rise to 2^6^=32 possible response curves and 32 corresponding AUCs. For every PERC *n* and drug *d* we constructed the AUC set:


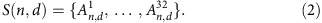


The parent line PC9-1 was assayed twice for each drug (each with two replicates per concentration). We denote these two replicate assays here by PC9-1r_1_ and PC9-1r_2_ and define a corresponding AUC set for PC9-1 by combining the corresponding AUC sets:


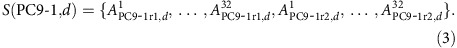


Then, the drug response for PERC *n* and drug *d* was characterized in terms of its difference from the PC9-1 response as quantified by the test statistic of the Student's *t*-test (two sample, unequal sample sizes and variances):


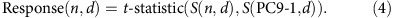


### Smoothed drug-response curves

The mean (across replicates) dose–response curves were fit to the sigmoidal form using a least-squares unweighted model (*lsqnonlin* in Matlab v2014a)^38^:


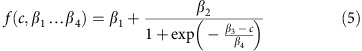


where *c* represents the concentration and *β*_1_… *β*_4_ are the parameters to be fit, subject to the constraints that *β*_1_>0, *β*_2_>0 and *β*_4_>0.

### Drug-category-response scores

We developed a statistical measure to prioritize specific PERC/drug-category combinations. In brief, we define ‘effective' drugs in terms of a response threshold, we look for drug categories enriched for effective drugs and then scan for the highest value of threshold for which a category remains enriched. We define effective, threshold and enriched below.

(1) *Effective drug.* For a given PERC *x* and response threshold *T*, we define a drug *d* to be effective if Response(*x*, *d*)>*T* for identifying drug sensitivity (and Response (*x*, *d*)<−*T* for identifying drug resistance).

(2) *Enriched drug category.* For a given PERC *x* and response threshold *T*, we quantify using the cumulative hypergeometric distribution, *H*, the extent to which a drug category *C* is enriched for effective drugs by:


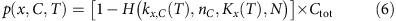


where *k*_*x,C*_(*T*) is the number of effective drugs in category *C*, *n*_*C*_ is the number of drugs in category *C* and *K*_*x*_(*T*) is the total number of effective drugs for PERC *x*, *N*=560 is the total number of drugs screened and *C*_tot_ is the total number of category (for Bonferroni-type correction of multiple classes being tested). Here *H*(*k*, *n*, *K*, *N*) calculates the cumulative hypergeometric distribution, with up to *k* successes in *n* draws, without replacement, from a finite population of size *N* that contains exactly *K* successes.

(3) *Max threshold for enriched category*. For a given PERC *x* and drug-category *C* we compute the maximum threshold *T* at which *p*(*x*, *C*, *T*)<*S*_*p*_. This is used as our drug-category-response score. In practice, we empirically chose *S*_*p*_=0.01 and varied the threshold *T* from 1 to 200 in steps of 1 to identify when the various categories fall out (Supplementary Fig. 3). To ensure that we had enough evidence to support our hits, we only counted *P* values supported by at least three responding drugs. We note that *p*(*x*, *C*, *T*) is not a monotonic function of *T*.

### Exome-seq analysis

Data were processed and aligned to the reference genome hg19 by BGI using Burrows-Wheeler alignment tool, BWA ALN. Somatic single-nucleotide variants (compared with PC9-1) were called using MuTect^40^ with default parameters. Somatic copy number variations (CNVs; compared with PC9-1) were called using ExomeCNV^41^ with default parameters to provide a specificity and sensitivity of 99.99%. CNVs with read ratios in the range 0.6<ratio<1.4 were filtered out.

Variants called reflect a difference in the genetic state between PC9-1 and the PERCs. These can either arise from (a) evolution of the PERCs in drug or (b) from the evolution of PC9-1, as it was being expanded out of drug to perform sequencing. Events of type b) manifest as differences from PC9-1 that are common to all PERCs and were found to be relatively rare. As our focus was on the evolution of PERCs, we dropped such events in Fig. 3a (where only a single NRAS deletion common to all PERCs was omitted).

### Relative drug-response score

If *A*_*n,d*_ denotes the AUC for the *n*th PERC when treated with drug *d*, then we measured a relative drug response in terms of the percentage difference from the mean PERC AUC (across all lines) of the drug


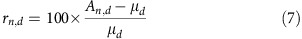


where 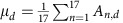. Here *A*_*n,d*_ was computed from the smoothed response curve (Fig. 3b and Supplementary Figs 5,6a and 7a).

### RPPA analysis

The output of the RPPA experiment was a matrix of values, each row corresponding to a cell line (PERC or PC9-1) and each column corresponding to an antibody. These data were analysed as follows:

1. *Normalization*: the data (which are in log 2) were linearized (by raising to power 2), each row (cell line) was divided by its median, each column (antibody) was divided by its median and the values were then converted back to log 2.
2. *Replicate averaging*: replicates for each cell line were averaged to generate cell line profiles.
3. For each cell line and antibody, the level of the corresponding antibody for PC9-1 was subtracted.
4. *Quality*: antibodies marked as Validated (V), Caution (C) or QC by MD Anderson RPPA Core Facility were used. (V): Pearson correlation coefficient between in-house RPPA data and western blot data is >0.7. (C): Pearson correlation coefficient between in-house RPPA data and western blot data is <0.7. (QC): antibody is suitable for cell line analysis but not tissue sample analysis.

### Code availability

Computer code used to generate Figs 1, 2, 3 is available as Supplementary Software 1–3.

## Additional information

**Accession codes:** Deposited at the Sequence Read Archive with accession number SRP068321.

**How to cite this article:** Ramirez, M. *et al.* Diverse drug-resistance mechanisms can emerge from drug-tolerant cancer persister cells. *Nat. Commun.* 7:10690 doi: 10.1038/ncomms10690 (2016).

## Supplementary Material

Supplementary Information Supplementary Figures 1-9 and Supplementary Table 1.

Supplementary Data 1 Annotation of Drugs used In Drug Screen

Supplementary Software 1 MATLAB code used in the generation of Fig1

Supplementary Software 2 MATLAB code used in the generation of Fig2

Supplementary Software 3 MATLAB code used in the generation of Fig3
