# Supplementary material for: Diverse drug-resistance mechanisms can emerge from drug-tolerant cancer persister cells
Source: Nat Commun. 2016 Feb 19;7:10690. doi: 10.1038/ncomms10690 (PMC4762880; doi:10.1038/ncomms10690)
Supplement: Supplementary Information — Supplementary Figures 1-9 and Supplementary Table 1. [file ncomms10690-s1.pdf]

Supplementary Fig 1

a)

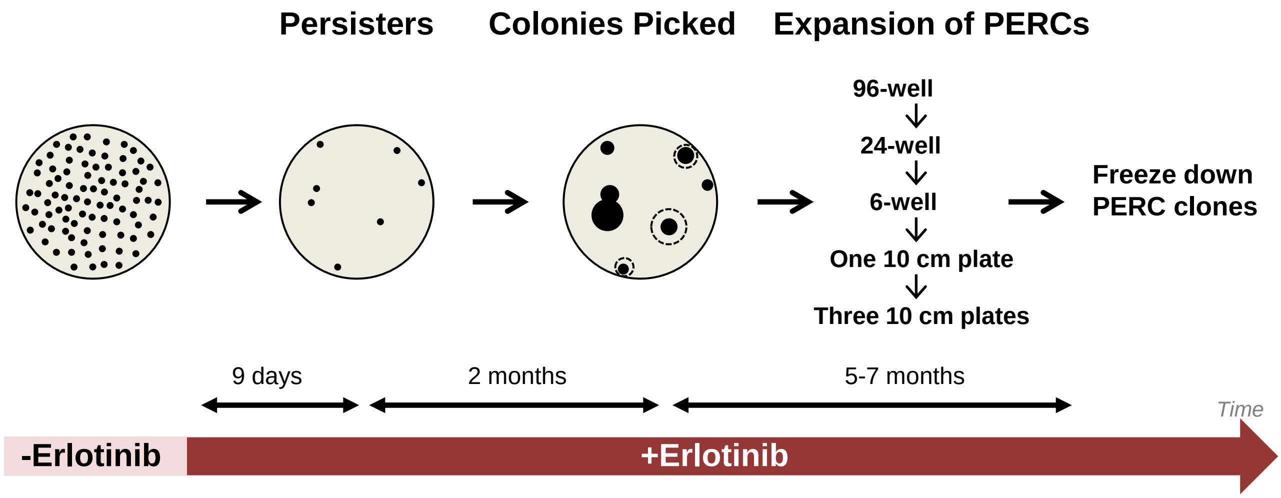

b)

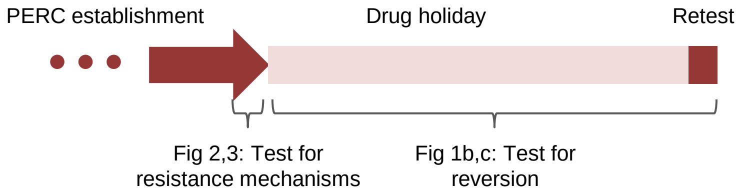

Supplementary Figure 1: Assessing PERC drug-resistance mechanisms *via* genetics and specific perturbations

a. Schematic representation of PERC isolation process. 1 X 10^5 cells were plated on several 10 cm plates, allowed to stabilize overnight then continuously treated with 2.5 µM erlotinib-containing media throughout the course of isolation and expansion. After 9 days, drug-tolerant persisters remained. Sizable colonies were visible 2 months after initial persister stage, and spatially separated colonies were picked and transferred to individual wells of a 96-well plate. Over the course of ~7 ± 1.5 months, selected colonies were expanded by transfer to larger cell culture vessels.

b. Schematic overview of experimental design and figures after establishment of PERCs.

Supplementary Fig 2

a)

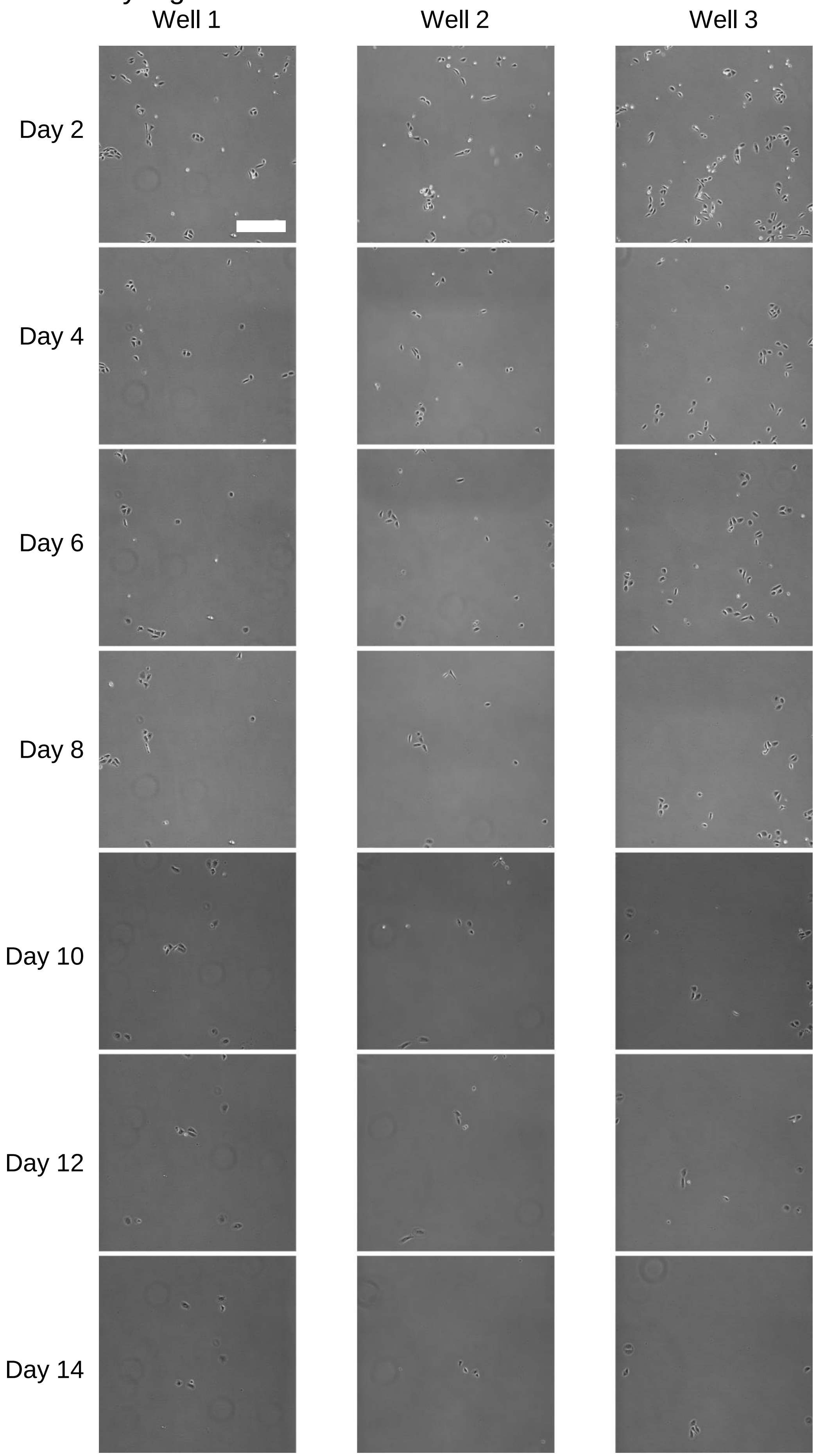

Supplementary Fig 2

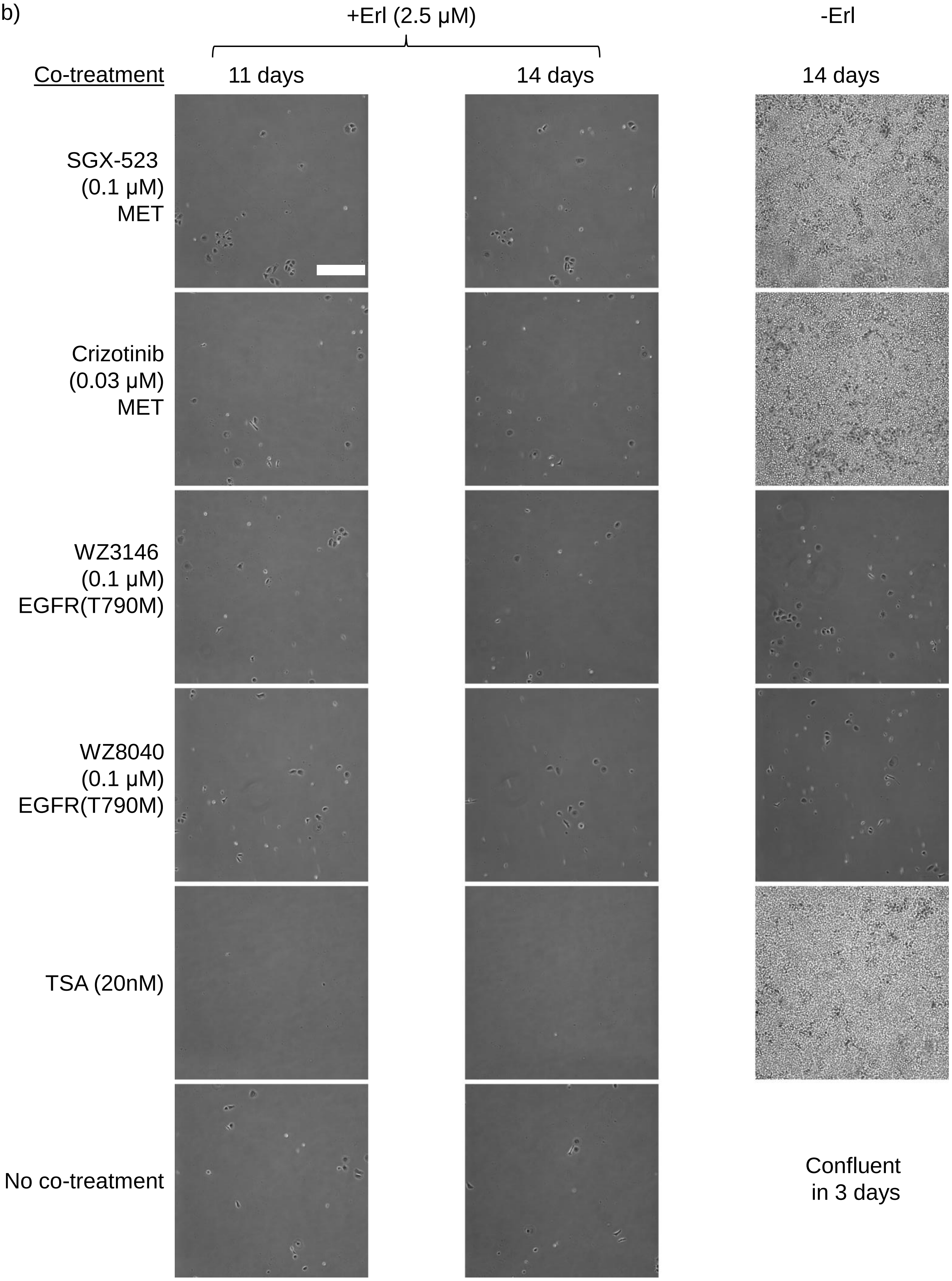

Supplementary Fig 2

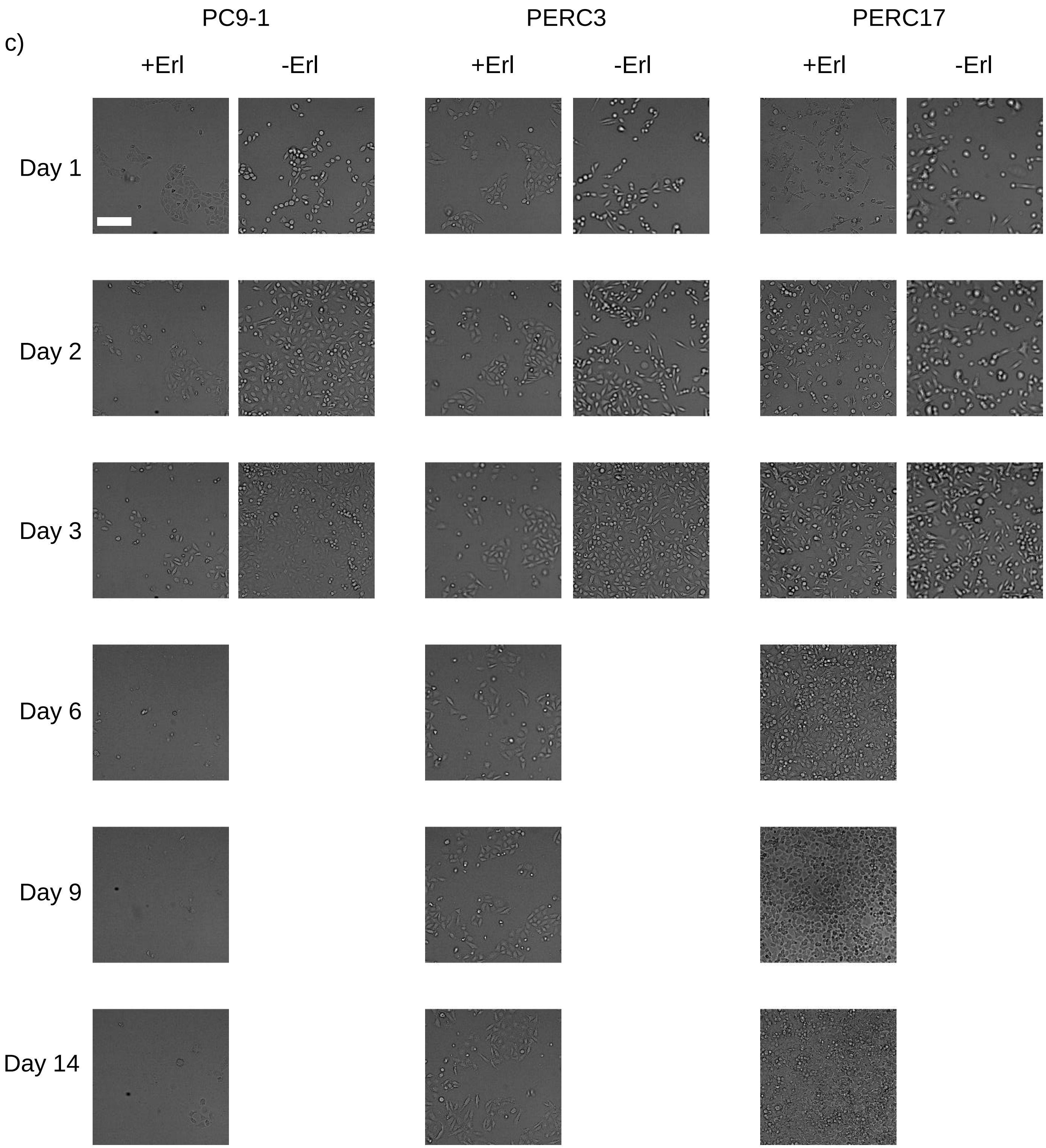

Supplementary Figure 2: Phase contrast and brightfield images of cells in drug treatment

a) PC9-1 cells enter a slow-growing drug-tolerant persister state when treated with erlotinib: PC9-1 cells were plated in a 6-well plate and treated with 2.5 µM erlotinib over the course of 14 days. Shown are sample phase contrast images from three replicate wells (columns) every 2 days (rows). Scale Bar is 0.16mm

b) Effect of pharmacological perturbations on the establishment of the persister state: PC9-1 cells were plated in a 6-well plate and treated with various drugs (rows, with drug name on left) either singly (right column) or in combination (left and middle columns) with 2.5 µM erlotinib over the course of 14 days. Shown are sample phase contrast images after either 11 or 14 days of drug treatment. Scale Bar is 0.16mm

c) Slow-growth response of PERC3 to erlotinib after long-term drug holiday:
Bright field images of PERC3 after 46 weeks of growth in drug-free media and subsequent re-treatment with 2.5 µM erlotinib (rows indicate specified days after erlotinib treatment and columns are cell line/drug combinations). Visual inspection reveals that the ratio of cells (+/- erlotinib) are comparable for PC9-1 and PERC3 after 3 days of drug treatment (and both ratios are lower than for PERC17) in agreement with the CellTiter-Glo result (Fig. 1c). However, in subsequent days with erlotinib treatment, while the PC9-1 population is dramatically reduced, PERC3 continues to grow (albeit more slowly than the other PERCs; non-treated plates become overgrown as can be seen from Day 3 images). Scale Bar is 0.16mm

Supplementary Fig 3

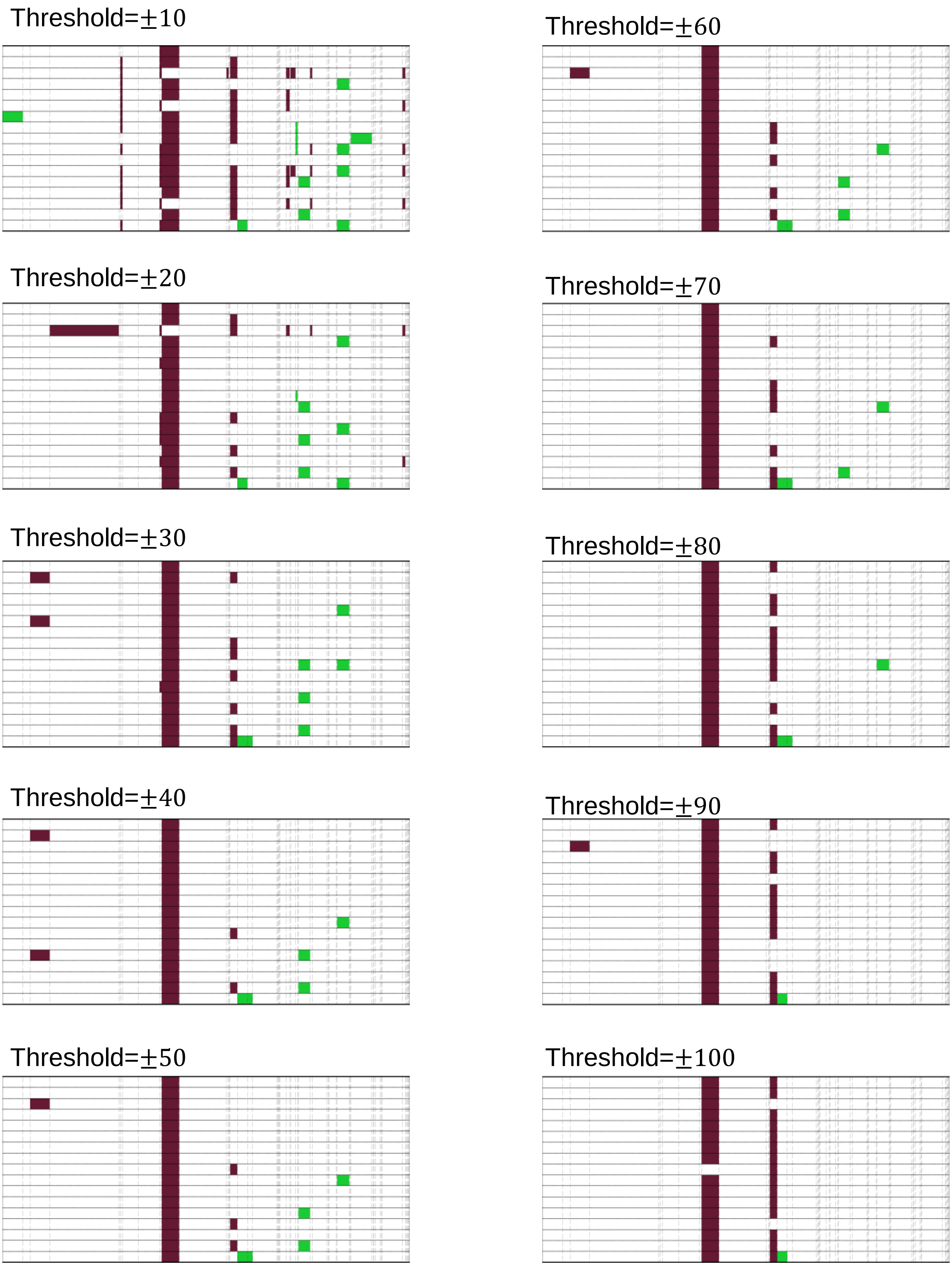

Supplementary Figure 3: Enrichment for strong response in drug categories as a function of threshold above which response is considered strong.

A PERC is deemed to show enrichment for strong response in a drug category if a higher than expected (Bonferroni corrected hyper-geometric p-value<0.01) number of drugs elicit a “strong response”; strong response is defined in terms of the drug-response score for a drug/PERC combination exceeding a specified threshold. Shown here is the effect of varying this threshold; each panel corresponds to a specified threshold value (each row is a PERC and columns are drugs). All drugs in a category are colored green/red for a PERC if the drug category is deemed to elicit strongly increased/decreased sensitivity as defined above.

Supplementary Fig 4

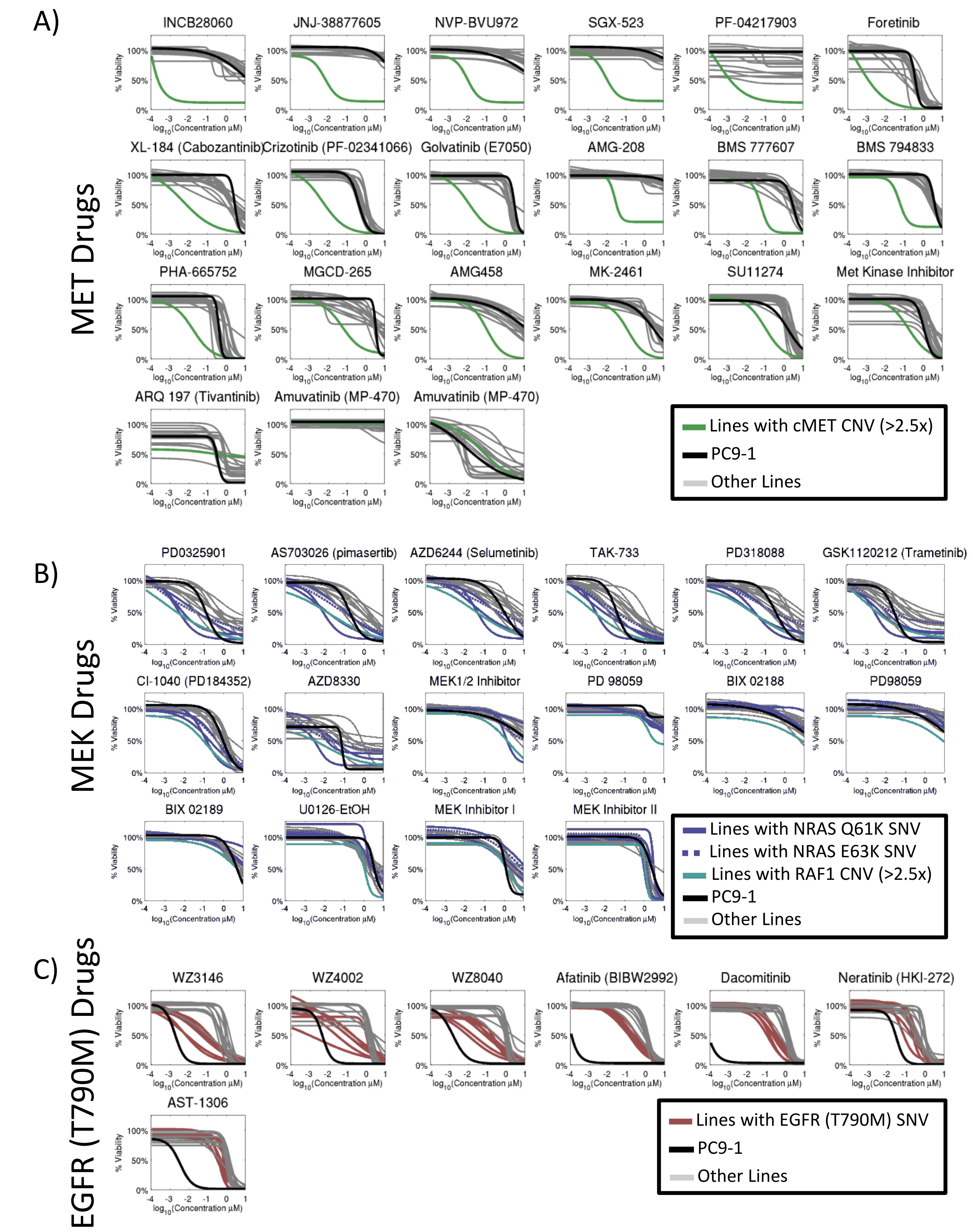

Supplementary Figure 4: PERCs drug response curves to MET, MEK and T790M targeting drugs.

Each plot represents shows dose-response curves for a given drug (as in Figs. 3c,e). x-axis: drug concentration; y-axis: titer-glo intensity with respect to a DMSO control. Note that in all cases, dose-response curves were generated using PERCs in erlotinib-containing media, and PC9-1 cells in drug-free media. For each PERC, viability was measured at six drug doses, with technical replicates (*n=2*) at each dose. Smoothed curves were constructed by fitting the mean viability at each dose to a sigmoidal function using an unweighted least squares fit (Methods). The PC9-1 curves are show in black. A) MET Drugs: PERC17 which has a cMET amplification is marked in green, while the other PERCs are marked in gray. B) MEK Drugs: Lines with mutations upstream of MEK are colored. PERCs 10,13,14 & 15 which have an NRAS mutation are marked in blue. PERC 16, which is the only PERC with a >2.5 fold RAF1 amplification is in Cyan. All other lines are marked in gray. C) T790M drugs: PERCs 1,4,5,6,7,8,9 with the T790M mutation are marked in red. All other lines are marked in gray.

Supplementary Fig 5

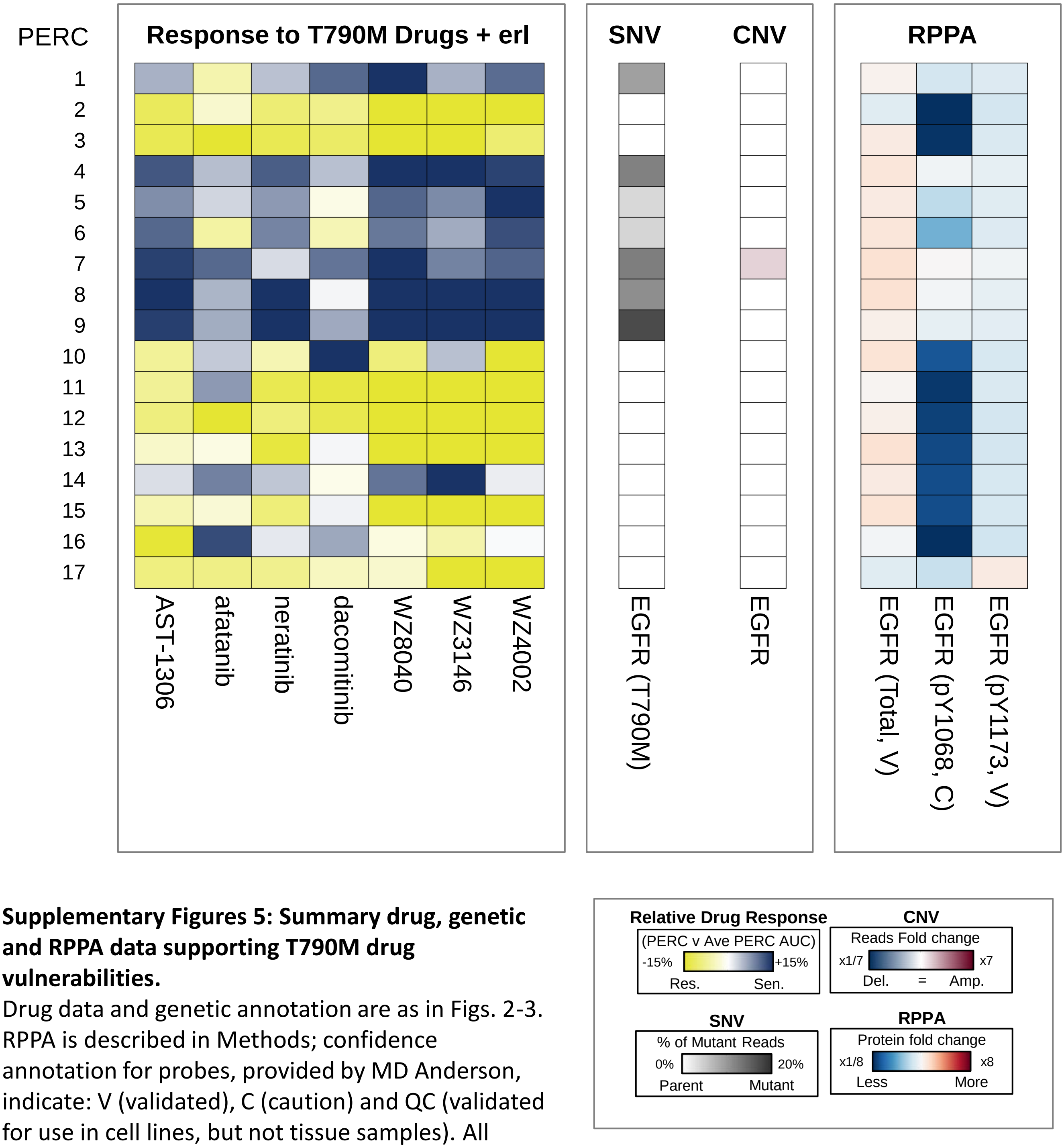

Supplementary Figures 5: Summary drug, genetic and RPPA data supporting T790M drug vulnerabilities.
Drug data and genetic annotation are as in Figs. 2-3. RPPA is described in Methods; confidence annotation for probes, provided by MD Anderson, indicate: V (validated), C (caution) and QC (validated for use in cell lines, but not tissue samples). All experiments were performed using PERCs in erlotinib-containing media.

Supplementary Fig 6

a)
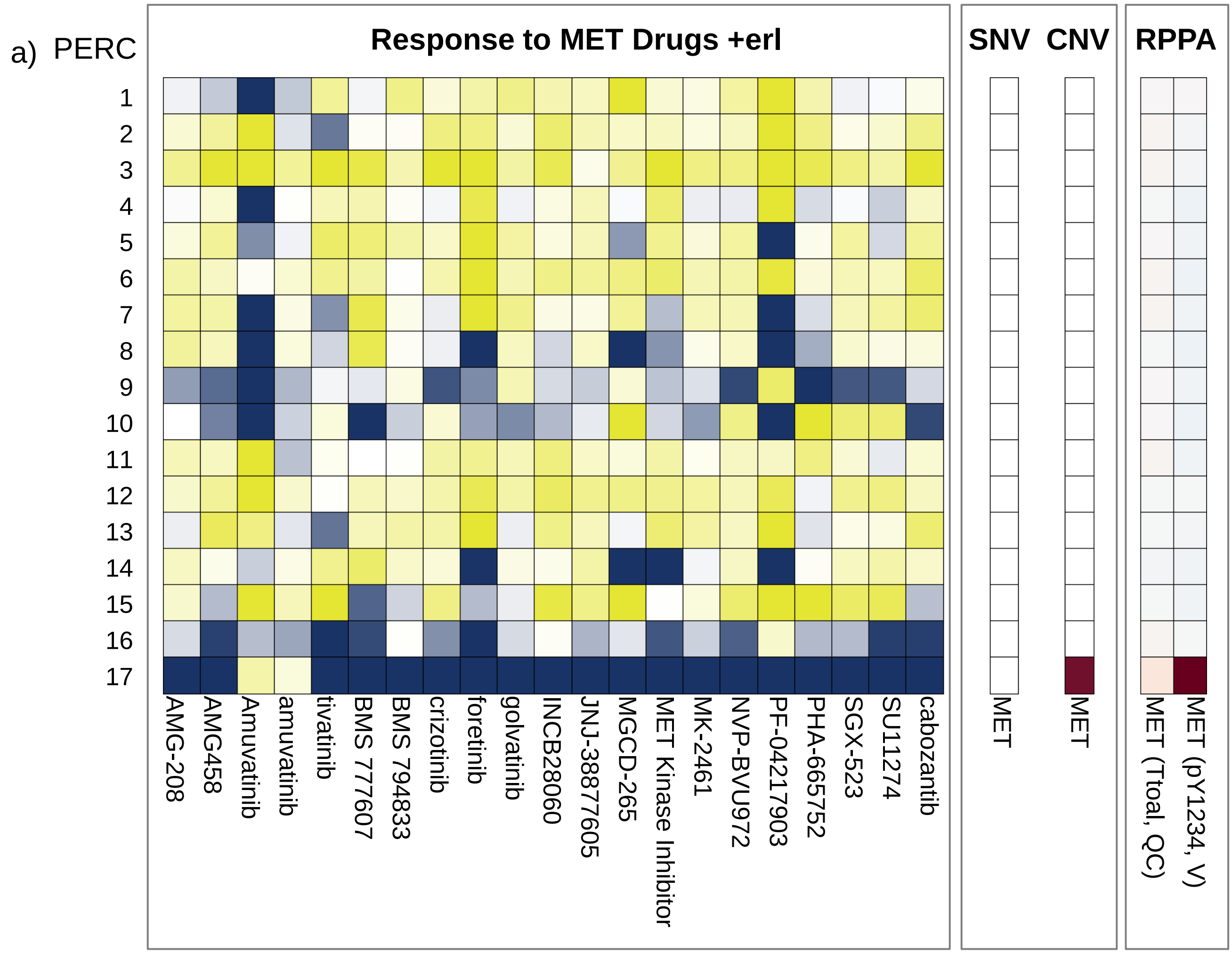

b)
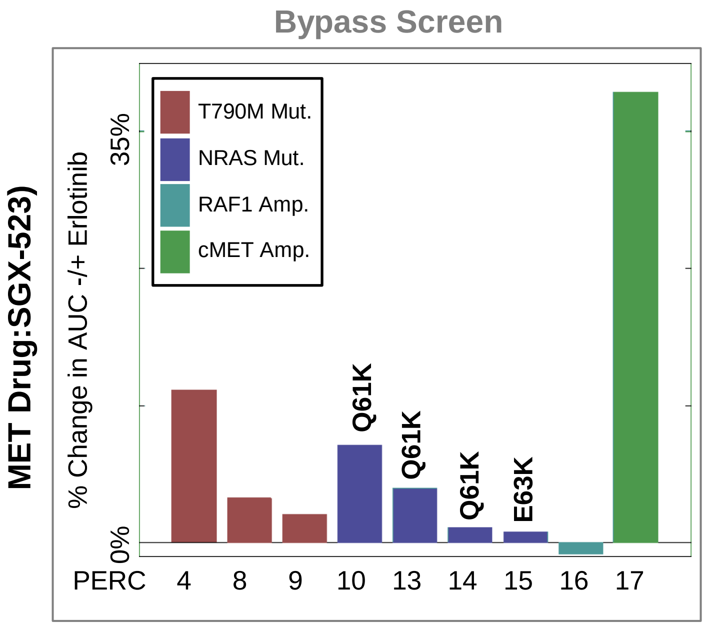

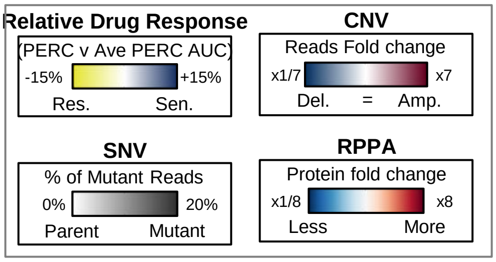

Supplementary Fig 6

c)

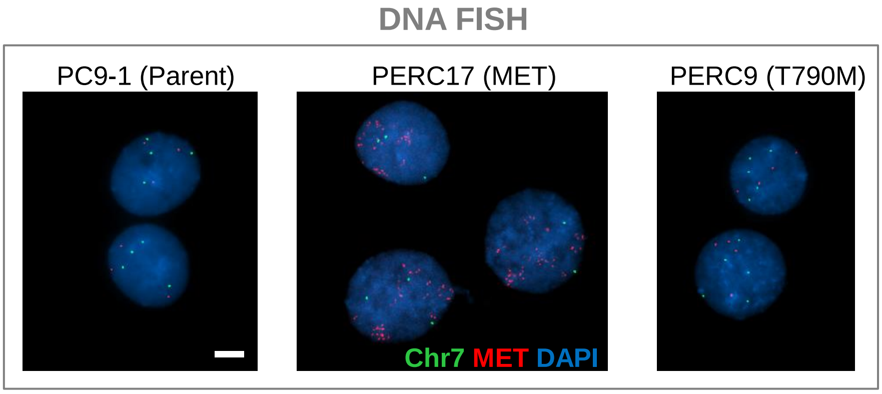

d)

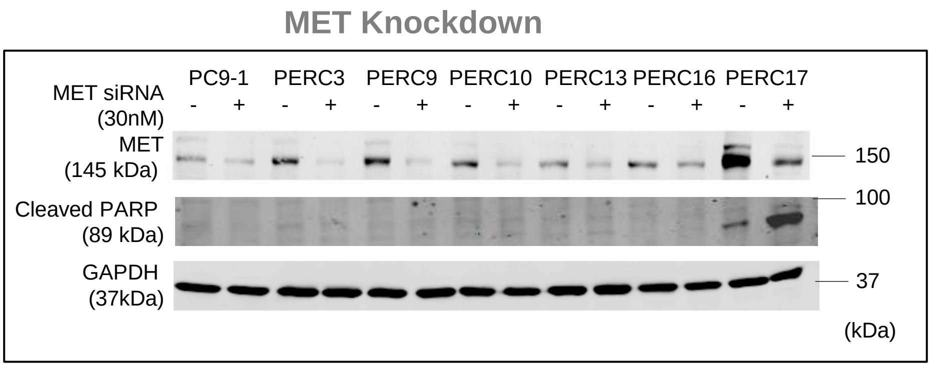

e)

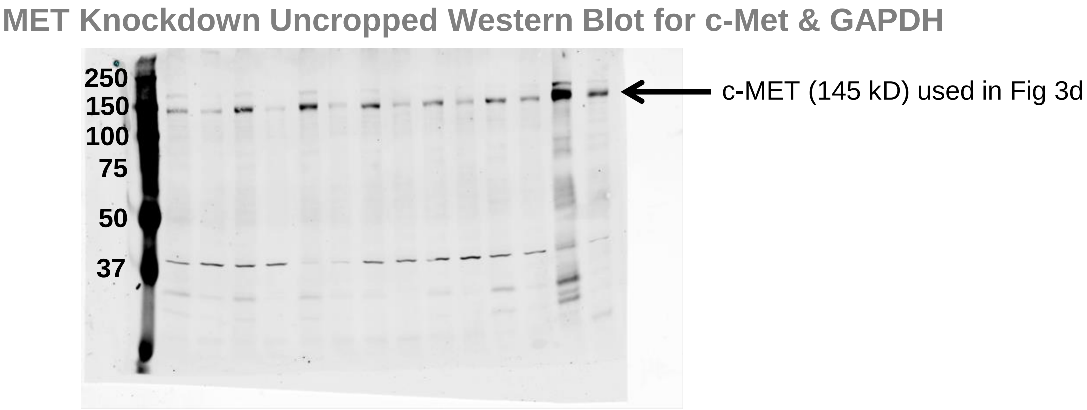

f)

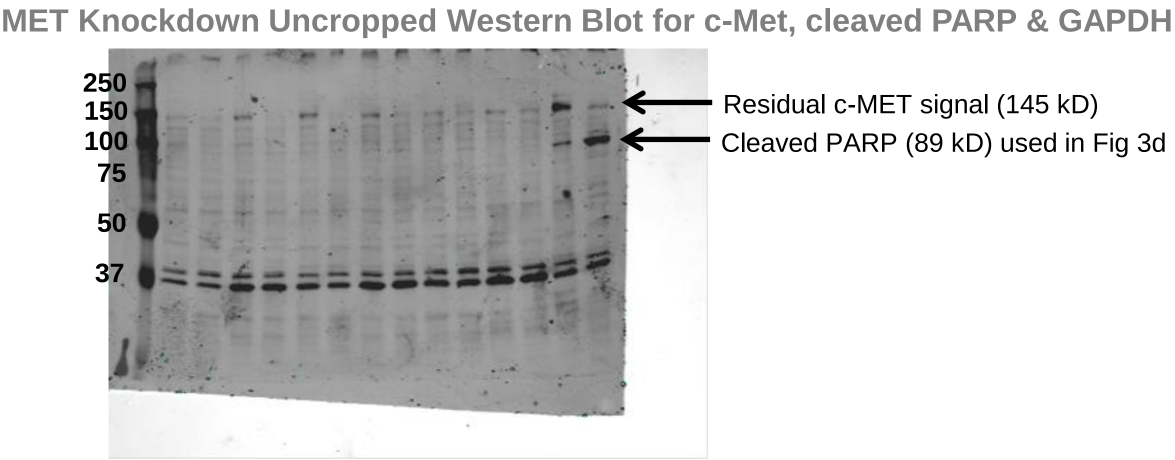

Supplementary Fig 6

MET Knockdown Uncropped Western Blot for GAPDH

g)

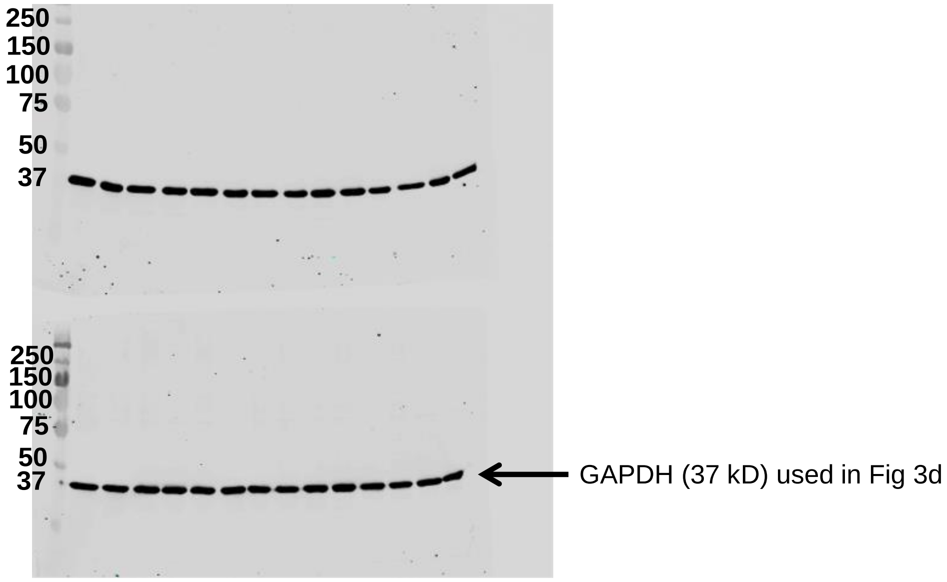

Supplementary Figures 6: Summary drug, genetic bypass, RPPA, FISH and knockdown data supporting MET drug vulnerabilities.

(a) Drug data and genetic annotation are as in Figs. 2-3. RPPA is described in Methods and Supplementary Fig 5. (b) Bypass screen is as in Fig 3f, but with a MET drug. (c) FISH assay for MET is described in Methods (scale bar is 10 microns). (d-g) Knockdown experiments and western blotting as in Fig 3d, with full uncropped blots shown. With the exception of the comparisons -/+ erlotinib, all experiments were performed using PERCs in erlotinib-containing media and PC9-1 cells in drug-free media.

Supplementary Fig 7

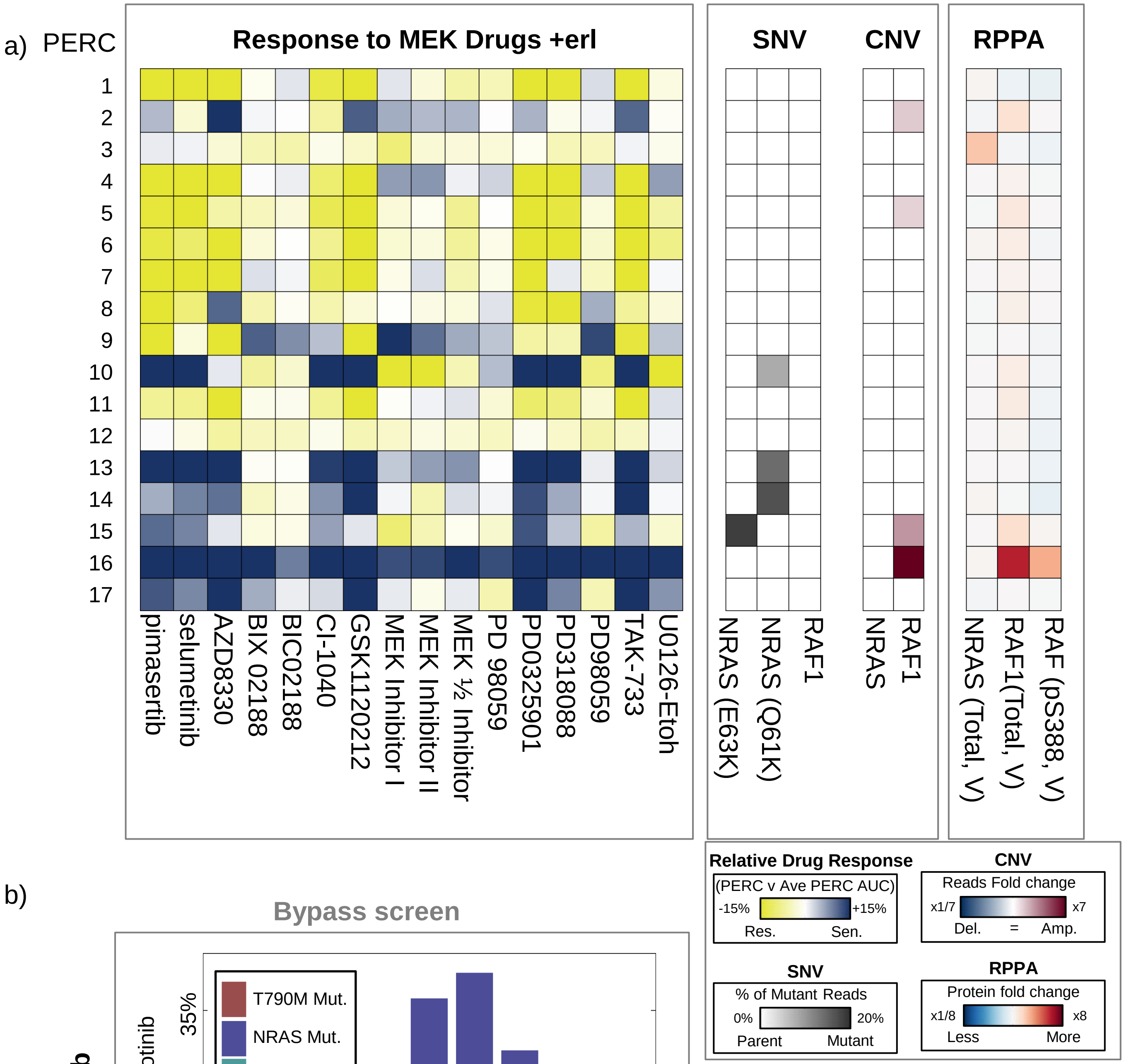

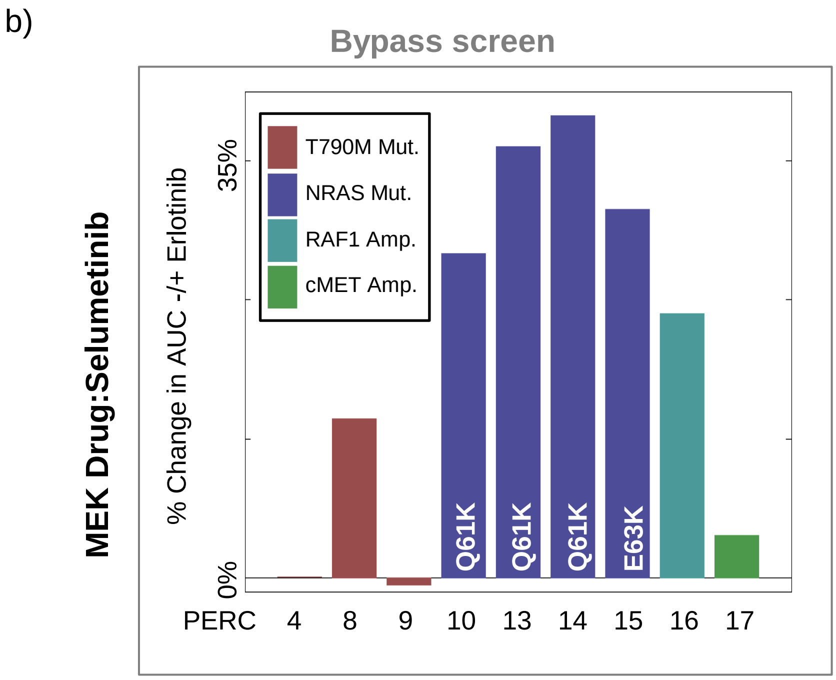

Supplementary Figures 7: Summary drug, genetic, RPPA and bypass data supporting vulnerability to MEK drugs
(a) Drug data and genetic annotation are as in Figs. 2-3. RPPA is described in Methods and Supplementary Fig 5. (b) Bypass screen is as in Fig 3f. With the exception of the comparisons -/+ erlotinib, all experiments were performed using PERCs in erlotinib-containing media, and PC9-1 cells in drug-free media.

Supplementary Fig 8

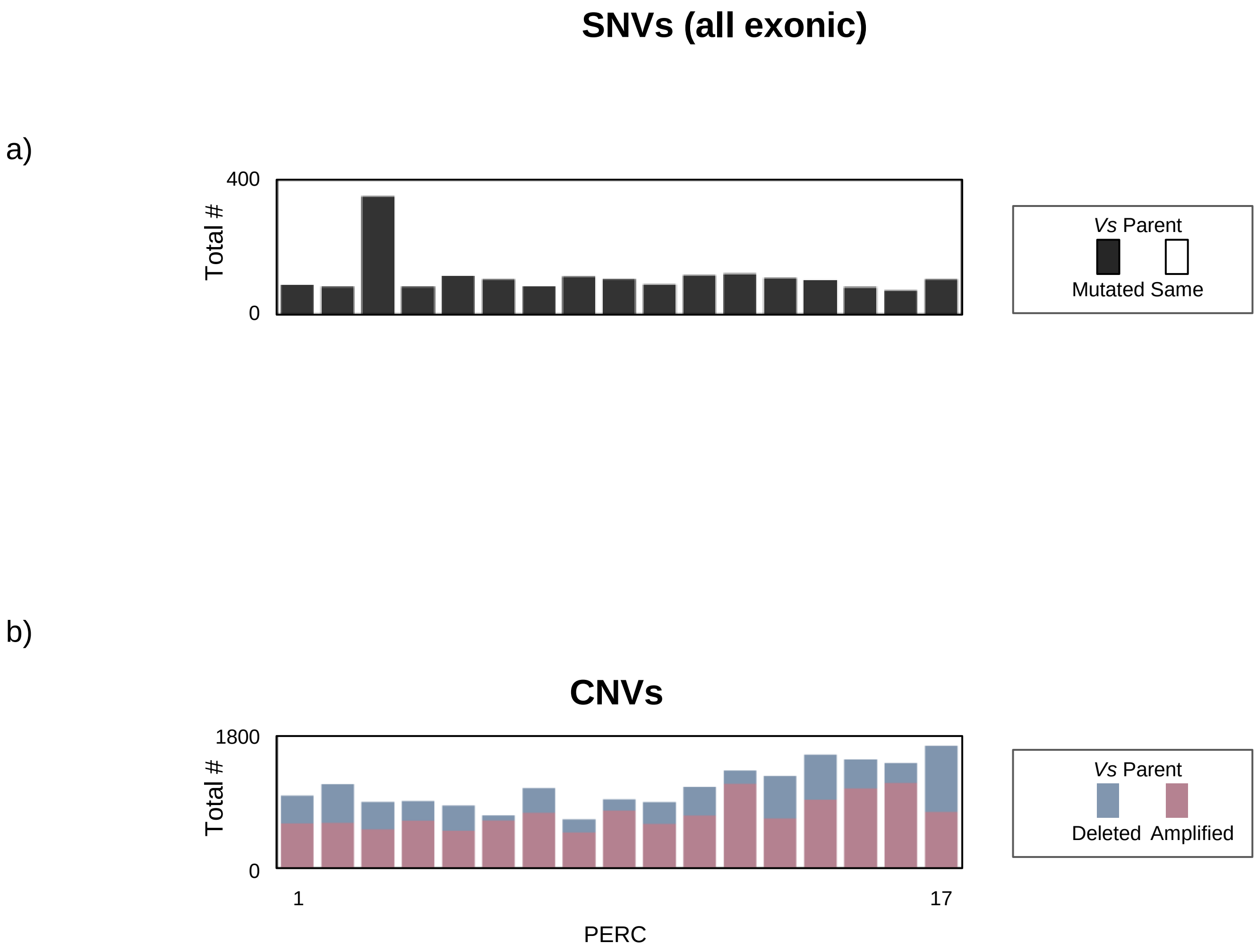

Supplementary Figure 8: Abnormally high number of SNV mutations for PERC3.
Bar plots show the total number of exonic nucleotides with SNVs (**a**) or genes with CNVs (**b**) per PERC

Supplementary Fig 9

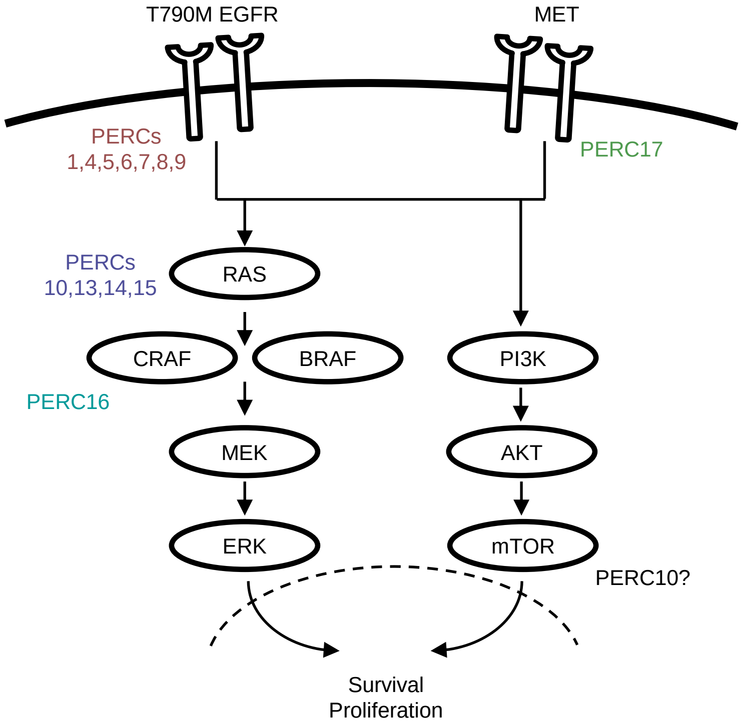

Supplementary Figure 9: Summary of observed erlotinib-resistance heterogeneity for PERCs. Our collection of PERCs acquired multiple mechanisms of erlotinib resistance: PERC17 harbored a MET amplification; PERCs 1,4,5,6,7,8,9 harbored the T790M mutation in EGFR; PERCs 10,13,14,15 harbored mutations in NRAS. PERC16 harbored a C-RAF (RAF1) amplification; and PERC10 showed drug-sensitivity to mTOR drugs, which could not be explained with genetic information.

Supplementary Table 1

CellLine	SNVs	CNVs
PERC1	EGFR(p.T790M)	
PERC2		RAF1(x1.5315),NF1(x0.37639)
PERC3		
PERC4	EGFR(p.T790M)	
PERC5	EGFR(p.T790M)	RAF1(x1.4251),NF1(x0.4061),KRAS(x0.58593),AKT1(x1.6197)
PERC6	EGFR(p.T790M)	
PERC7	EGFR(p.T790M)	EGFR(x1.4414)
PERC8	EGFR(p.T790M)	
PERC9	PIK3CA(p.E542K),EGFR(p.T790M)	
PERC10	NRAS(p.Q61K)	
PERC11	PIK3CB(p.E563K),BRAF(p.G466A)	HRAS(x1.5675)
PERC12		TSC1(x1.6241)
PERC13	NRAS(p.Q61K)	HRAS(x1.5983)
PERC14	NRAS(p.Q61K)	HRAS(x1.5779)
PERC15	NRAS(p.E63K)	RAF1(x2.2914)
PERC16		RAF1(x22.1433),HRAS(x1.5933)
PERC17		MET(x6.4958)

Supplementary Table 1: List of SNVs and CNVs displayed in Fig 3a.
